# Screening of Heavy Metal-Immobilizing Bacteria and Its Effect on Reducing Cd^2+^ and Pb^2+^ Concentrations in Water Spinach (*Ipomoea aquatic* Forsk.)

**DOI:** 10.3390/ijerph17093122

**Published:** 2020-04-30

**Authors:** Tiejun Wang, Xiaoyu Wang, Wei Tian, Lunguang Yao, Yadong Li, Zhaojin Chen, Hui Han

**Affiliations:** 1 Collaborative Innovation of Water Security for the Water Source Region of Mid-line of the South-to-North Diversion Project of Henan Province, College of Agricultural Engineering, Nanyang Normal University, Nanyang 473061, China; liuyangkun@nynu.edu.cn (T.W.); tcd530@nynu.edu.cn (X.W.); lunguangyao@nynu.edu.cn (L.Y.); 2 State Key Laboratory of Biocatalysis and Enzyme Engineering, School of Life Sciences, Hubei University, Wuhan 430062, China; hbchen7112@hubu.edu.cn; 3 Nanjing Institute of Environmental Sciences, Ministry of Ecology and Environment, Nanjing 210042, China; tianwei@nies.org

**Keywords:** heavy metal-immobilizing bacteria, Cd^2+^ and Pb^2+^, water spinach, immobilization, community composition

## Abstract

Microbial immobilization is considered as a novel and environmentally friendly technology that uses microbes to reduce heavy metals accumulation in plants. To explore microbial resources which are useful in these applications, three water spinach rhizosphere soils polluted by different levels of heavy metals (heavy pollution (CQ), medium pollution (JZ), and relative clean (NF)) were collected. The community composition of heavy metal-immobilizing bacteria in rhizosphere soils and its effects on reducing the Cd^2+^ and Pb^2+^ concentrations in water spinach were evaluated. Four hundred strains were isolated from the CQ (belonging to 3 phyla and 14 genera), JZ (belonging to 4 phyla and 25 genera) and NF (belonged to 6 phyla and 34 genera) samples, respectively. In the CQ sample, 137 strains showed a strong ability to immobilize Cd^2+^ and Pb^2+^, giving Cd^2+^ and Pb^2+^ removal rates of greater than 80% in solution; *Brevundimonas*, *Serratia*, and *Pseudoarthrobacter* were the main genera. In total, 62 strains showed a strong ability to immobilize Cd^2+^ and Pb^2+^ in the JZ sample and *Bacillus* and *Serratia* were the main genera. A total of 22 strains showed a strong ability to immobilize Cd^2+^ and Pb^2+^ in the NF sample, and *Bacillus* was the main genus. Compared to the control, *Enterobacter bugandensis* CQ-7, *Bacillus thuringensis* CQ-33, and *Klebsiella michiganensis* CQ-169 significantly increased the dry weight (17.16–148%) of water spinach and reduced the contents of Cd^2+^ (59.78–72.41%) and Pb^2+^ (43.36–74.21%) in water spinach. Moreover, the soluble protein and Vc contents in the shoots of water spinach were also significantly increased (72.1–193%) in the presence of strains CQ-7, CQ-33 and CQ-169 compared to the control. In addition, the contents of Cd and Pb in the shoots of water spinach meet the standard for limit of Cd^2+^ and Pb^2+^ in vegetables in the presence of strains CQ-7, CQ-33 and CQ-169. Thus, the results provide strains as resources and a theoretical basis for the remediation of Cd- and Pb-contaminated farmlands for the safe production of vegetables.

## 1. Introduction

With the rapid development of urbanization, environmental problems such as heavy metal accumulation are increasing in farmland areas because of the large amounts of pollutants being discharged and increased use of agricultural inputs, such as fertilizer [1,2,3]. Cadmium (Cd^2+^) and lead (Pb^2+^) are regarded as the most toxic environmental pollutants, as they display the most profound mobility in the soil environment and cannot be degraded by microorganisms [4]. Water spinach (*Ipomoea aquatic* Forsk.) is a dominant global vegetable [5]. Excessive Cd^2+^ and Pb^2+^ accumulation in water spinach can reduce yields and cause potential threats to human health through the food chain [6,7,8]. Therefore, to ensure the safety of agricultural products, it is urgent that we carry out the bioremediation of farmland to remove medium and light heavy metals exceeding standard levels [9,10].

Currently, in situ passivation is a commonly used technology for the farmland remediation of medium and light heavy metal pollution, and mainly includes chemical passivation remediation and microbial immobilization remediation [11,12,13]. The use of biochar and other chemical passivators immobilizes heavy metals in soil and reduces the absorption of heavy metals by plants [14,15]. However, the long-term use of chemical passivators causes soil hardening, alterations in the physical and chemical properties of soil, and decreases in fertility [16]. Microbial-immobilized remediation technology refers to using soil microorganisms to immobilize heavy metals, causing heavy metals to precipitate or be adsorbed and fixed in the soil, reducing their absorption by plants [17]. In recent years, heavy metal-immobilizing bacteria have been widely studied and applied as excellent heavy metal passivators. Heavy metal-immobilizing bacteria not only immobilize heavy metals, alter the existing state of heavy metals in soil, and reduce the absorption of heavy metals by crops, but also promote the growth of crops and improve the quality of crops [13,18]. Bacteria increased the growth and heavy metal resistance of vegetables by producing indole acetic acid (IAA), siderophores, 1-aminocyclopropane-1-carboxylate deaminase, and arginine decarboxylase. Moreover, heavy metal-immobilizing bacteria increased vegetables’ competitiveness and improved the quality of vegetables [19]. Many scholars had proved that heavy metal-immobilizing bacteria can prevent the absorption of heavy metals by crops such as rape [20], rice [21] and radish [22]. However, there were few reports on the use of heavy metal-immobilizing bacteria to reduce Cd and Pb concentrations in water spinach. Therefore, it is necessary to continue to tap microbial resources and screen heavy metal-immobilizing bacteria that can prevent the absorption of Cd^2+^ and Pb^2+^ by water spinach.

Long-term high concentrations of heavy metal damage microorganisms in the soil to different degrees and affect the distribution of the microbial population [23]. Additionally, to cope with stress caused by heavy metals, microorganisms have evolved mechanisms to overcome toxicity, including metal reduction, cell permeability reduction, and extracellular isolation [24]. Therefore, heavy metal-immobilizing bacteria screened from in situ heavy metal-contaminated plant rhizosphere soil show better environmental adaptability and higher ability for preventing crops from absorbing heavy metals [25]. Although high-throughput sequencing had been used to study the distribution of microbial populations under different heavy metal pollution conditions [23,26], few studies have examined the community differences of culturable bacteria and the proportion of heavy metal-immobilizing bacteria in water spinach rhizosphere soil with different levels of heavy metal pollution.

Therefore, the purposes of this work are: (1) Effects of long-term Cd- and Pb-pollution on culturable bacteria and heavy metal-immobilizing bacteria community in rhizosphere soil of water spinach were studied; (2) Screening the heavy metal-immobilizing bacteria which can prevent water spinach from absorbing Cd^2+^ and Pb^2+^. Three water spinach rhizosphere soil samples with different levels of Cd- and Pb-pollution were collected. The community composition of culturable bacteria in the water spinach rhizosphere soil was compared using culturable separation technology. The proportions of heavy metal-immobilizing bacteria in water spinach rhizosphere soils and its effects on immobilizing Cd^2+^ and Pb^2+^ were examined. In addition, the effects of the strains on the water spinach biomass, soluble protein and the vitamin C (Vc) contents, and heavy metal uptake were also investigated. Thus, the results provide strains as resources and a theoretical basis for the remediation of Cd- and Pb- contaminated farmlands for the safe production of vegetables.

## 2. Materials and Methods

### 2.1. Sample Collection and Analysis

**Figure 1:**
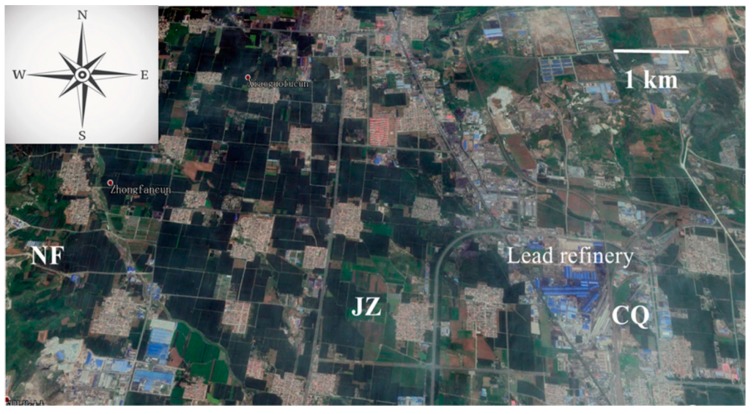
Sample sites of water spinach rhizosphere soil.

Henan Yuguang Gold lead Co., Ltd. is located in the suburb of Jiyuan City, Henan Province (35°13′N, 112°54′E) in China. The company is mainly engaged in the smelting and import and export trade of electrolytic lead, silver, gold, and other non-ferrous metals and precious metal products. Because of long-term sewage release by this company, the contents of Pb^2+^ and Cd^2+^ in the local soil and water greatly exceeded standard limits. Three water spinach rhizosphere soil samples, next to the company area (CQ), farmland near Jiazhuang village 3 km away from the company (JZ), and farmland near Nanfan village 6 km away from the plant (NF), were collected, respectively (Figure 1). The soil with an area of 10 cm × 10 cm and a depth of 0-15 cm around the water spinach (including water spinach) was excavated by a sampling shovel. Soil particles adhering tightly to the roots (constituting the rhizosphere soil) of the water spinach were gathered. Each soil sample contained three replicates. When the soil sample was dried at room temperature and sieved 100 mm, the physical and chemical properties were determined. Two grams of dry soil particles were balanced with 10 mL of deionized water for 30 min before its pH was measured by the use of a pH meter. Levels of organic matter in the rhizosphere soils were determined by the dichromate wet oxidation procedure [27]. The total N (TN) content of the rhizosphere soils was tested with the Kjeldahl approach [28]. Two grams of rhizosphere soil was digested with a catalyst mixture (CuSO_4_+/K_2_SO_4_+Se) and sulfuric acid (96%) at 350 °C. The solution resulting from the digestion was distilled with NaOH. The generated ammonia was condensed in H_3_BO_3_ solution, and the level of ammonia was determined through titrimetric analysis with sulfuric acid. The level of total phosphorus (TP) was subjected to colorimetric measurements at 660 nm using the molybdate ascorbic acid method contributed by Watanabe and Olsen [29]. The level of total potassium (TK) in the rhizosphere soils was measured by the flame photometry method after melting with sodium hydroxide and reaction at 720 °C for 20 min. Diethylenetriaminepentaacetic acid (DTPA) extract solution was added to the soil sample at the ratio of 2.5:1 (solution: soil) for 4 h, then the supernatant solution was digested by electrothermal digestion. The contents of DTPA-extractable Cd and Pb in the extract solution were determined by inductively coupled plasma atomic emission spectrometry (ICP-AES) (SHIMADZ ICPE-9820, Japan) [30].

### 2.2. Isolation of Culturable Bacteria from Water Spinach Rhizosphere Soil

Fresh soil samples (1.0 g) of CQ, JZ, and NF were placed in a triangular flask containing 50 mL of sterile deionized water and cultured for 30 min with shaking at 150 rpm and 30 °C. Next, 1 mL of the soil suspension was diluted to 10^−4^, 10^−5^, and 10^−6^ by gradient dilution and coated onto LB medium plates. After incubation for 3 days at 30 °C, the total number of culturable bacteria in the three samples was calculated by counting single colonies on all plates. According to the colony size, edge shape, color, and other indicators, colonies were selected from the CQ, JZ, and NF samples for purification and preservation, respectively. Genomic DNA of those bacteria strains were extracted using a commercial kit according to the manufacturer’s instructions. The primers 27F (5′- AGAGTTTGATCMTGGCTCAG -3′) and 1492R (5′-TACGGYTACCTTGTTACGACTT-3′) were used for amplification of 16S rRNA gene. The PCR reaction was completed as described by Teng et al. [19]. The amplified products were sent to Shanghai Biological Engineering Company (Shanghai, China) for sequencing and the sequence obtained was compared with those available in the EzBioCloud server (http://www.ezbiocloud.net/).

### 2.3. Determination of Cd and Pb Adsorption Capacity of the Strains

To determine the Cd^2+^ and Pb^2+^ removal rates by the strains, LB liquid media containing 5 mg L^−1^ Cd^2+^ (as Cd(NO_3_)_2_) and 20 mg L^−1^ Pb^2+^ (as Pb(NO_3_)_2_) (based on the concentration of available Cd and Pb in the CQ sample) were prepared. The suspension of tested strains, for which the OD_600_ value was adjusted to 1.0, was inoculated into a flask containing 50 mL LB liquid medium and cultured at 30 °C with shaking at 180 rpm for 48 h. At the end of culture, 5 mL of fermentation broth was centrifuged for 5 min at 5000 rpm. The levels of Cd^2+^ and Pb^2+^ in the supernatant were quantified by ICP-AES. Treatments without inoculation were performed to determine the initial concentrations of water-soluble Cd^2+^ and Pb^2+^ in the solution. The removal efficiency of Cd^2+^ and Pb^2+^ by each strain were calculated as follows:

(1) $$\mathrm{Heavy}\;\mathrm{metal}\;\mathrm{removal}\;\mathrm{efficiency}\;\%=\frac{C_{0}-C_{s}}{\;C_{0}}\times 100\%$$

where *C*_0_ represents the initial concentration of Cd^2+^ and Pb^2+^ and *C*s represents the final concentration of Cd^2+^ and Pb^2+^.

### 2.4. Determination of Biological Characteristics of Heavy Metal-Immobilizing Strains

The minimal inhibitory concentrations (MICs) of Cd^2+^ and Pb^2+^ were determined by the continuous gradient plate method [31]. The tested strains were inoculated onto an LB plate containing different concentrations of Cd (100, 200, 300, 400, 500, 600, and 700 mg L^−1^) and Pb (500, 1200, 1500, 1800, 2000, 2200, and 2500 mg L^−1^) and cultured for 3 days at 30 °C. Colonies that grew on the plate were transferred to an LB plate with the corresponding heavy metal concentration and cultured for 3 days at 30 °C. The indole-3-acetic (IAA) produced by the strains was quantified using high performance thin-layer chromatography (HPLTC) [32]. Siderophores produced by the strains were quantified using the chrome azurol-s analytical method [33].

### 2.5. Hydroponic Experiment

A hydroponic experiment was designed according to the method of Zornoza et al. [34] with some modifications. Several full-grained water spinach (*Ipomoea aquatic* Forsk.) seeds were surface-disinfected with 75% ethanol for 3 min, rinsed with sterile water 3 times, placed on a water agar (0.4%) plate, and cultured at 30 °C. When the seeds of water spinach were about to germinate, immersing them in the bacterial suspension (preparation of bacterial suspension: function strains were grown in LB medium. Cells in the stationary phase were collected by centrifugation at 3000× *g* for 10 min and resuspended at 1 × 10^8^ cells mL^−1^ in sterile distilled water). As a control, inoculation was performed with no bacteria. After the water spinach seeds had grown two leaflets, ten water spinach seedlings of the same size were transplanted into a pot (the upper layer was filled with 500 g quartz sand to fix the plant crops and the lower layer contained Hogland nutrient solution (945 mg L^−1^ Ca (NO_3_)_2_, 607 mg L^−1^ KNO_3_, 115 mg L^−1^ NH_4_·H_2_PO_4_, 493 mg L^−1^ MgSO_4_·7H_2_O, 2.13 mg L^−1^ MnSO_4_, 0.08 mg L^−1^ CuSO_4_, 0.22 mg L^−1^ ZnSO_4_, 2.86 mg L^−1^ H_3_BO_3_, 0.02 mg L^−1^ H_2_MoO_4_, 40 mg L^−1^ chelated iron). When the water spinach seedlings reached 5 cm high, Cd^2+^ and Pb^2+^ were added to the nutrient solutions to concentrations of 5 mg L^−1^ Cd and 20 mg L^−1^ Pb. The pots were placed in a humid room at Nanyang Normal University experiment station. The indoor temperature was 23 ± 3 °C. The plants were harvested on the 30th day after the heavy metals inoculation.

### 2.6. Analysis of Samples

The cultivated water spinach was removed from the pot and divided into the roots and shoots. The roots were soaked in 0.01 mol L^−1^ EDTA-2Na solution for 10 min to remove heavy metals adsorbed on the root surface. The fresh water spinach samples were divided into two parts, one for the determination of soluble protein and the vitamin C (Vc) contents and the other for the determination of dry weight and heavy metal contents. The 2,6-dichlorophenolindophenol sodium salt titrimetric method [35] was used to analyze the Vc contents of the shoots of water spinach. The soluble protein content of the shoots of water spinach was measured according to the method of [36]. The dry weights of roots and shoots were measured with an electronic scale. Then, the dried water spinach roots and shoots were ground with a grinder, and 0.5 g was used for microwave digestion. The contents of Pb^2+^ and Cd^2+^ in the digestion solution were determined by ICP-AES [30].

### 2.7. Statistical Analysis

One-way analysis of variance and Tukey’s test (*p* < 0.05) were used to compare the averages of dry weight, Cd^2+^ and Pb^2+^ contents in water spinach, soluble protein and the vitamin C contents in the shoots of water spinach in the presence of heavy metal-immobilizing bacteria with those of the controls. Statistical analyses were performed using SAS 8.2 (Statistical Analysis System, Cary, NC, USA). The experimental data were first tested for normality and homogeneity of variance, and the data conforming to normal distribution were expressed by mean ± standard deviation (x ± s).

## 3. Results

### 3.1. Physical and Chemical Properties of the Soil Samples

Three samples of water spinach rhizosphere soil were collected from farmland near Henan Yuguang Gold Lead Co., Ltd. in May 2019. The basic physical and chemical properties of the three soil samples are shown in Table 1. The contents of DTPA-extractable Cd (4.35 mg kg^−1^) and Pb (22.5 mg kg^−1^) in CQ were significantly higher than those in JZ and NF. According to the environmental quality standards for soils in China (GB15618-2018), the pollution degree of heavy metals in farmland soil was divided into five grades (I–V, corresponding to clean, relative clean, light pollution, medium pollution, and heavy pollution, respectively). Thus, the Cd and Pb pollution levels in CQ were at level V, in JZ were at level IV, and in NF were at level II. The number of bacteria in NF (1.28 × 10^6^ CFU g^−1^) was higher than those in JZ (1.65 × 10^4^ CFU g^−1^) and CQ (6.4 × 10^3^ CFU g^−1^). These results demonstrated that a higher concentration of heavy metals was associated with a smaller number of culturable bacteria in the rhizosphere of water spinach. The high concentration of heavy metals in soil inhibits the growth and reproduction of bacteria.

### 3.2. Effects of Different Contents of Cd and Pb on Culturable Bacterial Communities in Water Spinach Rhizosphere Soil

**Figure 2:**
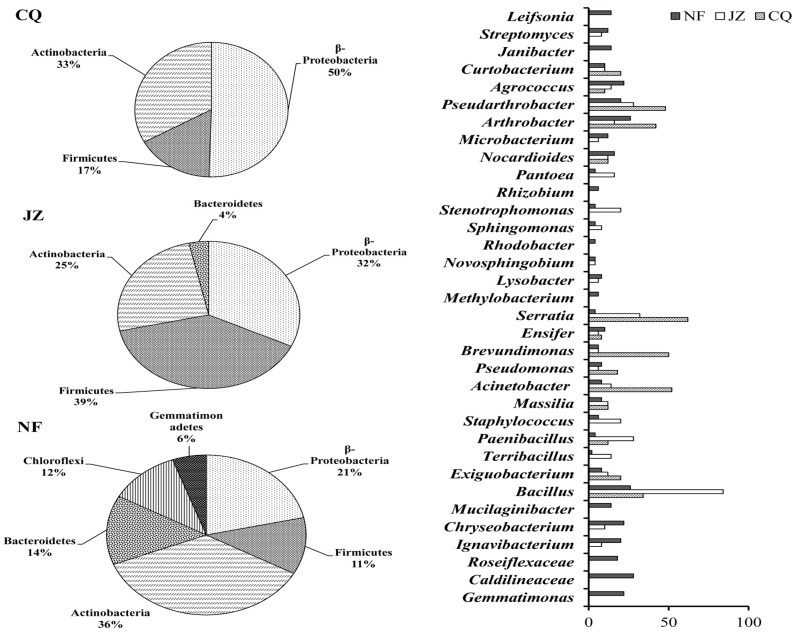
Differences of culturable bacteria in water spinach rhizosphere soil at the phylum and genus levels. CQ: farmland next to the company area; JZ: farmland near Jiazhuang village 3 km away from the company; NF: farmland near Nanfan village 6 km away from the company.

Culturable bacterial strains were isolated from fresh soil samples of CQ, JZ, and NF by the dilution coating plate method. Four hundred single colonies were selected from the plates for each sample. The community structure of culturable bacteria in each sample is shown in Figure 2. The 400 bacteria in the CQ samples belonged to 3 phyla and 14 genera. *β-*Proteobacteria was the dominant phylum, accounting for 50%, Actinobacteria accounted for 33%, and Firmicutes accounted for 17%. *Acinetobacter*, *Brevundimonas*, *Serratia*, *Arthrobacter*, and *Pseudarthrobacter* were the dominant genera, with more than 40 strains in these genera. In the JZ samples, the 400 bacteria belonged to 4 phyla and 25 genera. Firmicutes was the dominant phylum, accounting for 39%, *β-Proteobacteria* for 32%, Actinobacteria for 25%, and Bacteroidetes for 9%. Bacillus was the dominant genus, accounting for 84 strains. In the NF samples, the 400 bacteria belonged to 6 phyla and 34 genera. Actinobacteria was the dominant phylum, accounting for 36%, *β-Proteobacteria* for 21%, Bacteroidetes for 14%, Chloroflexi for 12%, Firmicutes for 11%, and Gemmatimona for 6%. *Caldilineaceae*, *Chryseobacterium*, *Bacillus*, *Arthrobacter*, *Pseudarthrobacter*, and *Agrococcus* were the dominant genera. The results showed that high concentrations of heavy metals reduced the number of bacteria taxa in the rhizosphere soil of water spinach at the phylum and genus levels and reduced bacterial diversity.

### 3.3. Comparison of Cd^2+^ and Pb^2+^ Removal Ability by Culturable Bacteria in Water Spinach Rhizosphere

**Figure 3:**
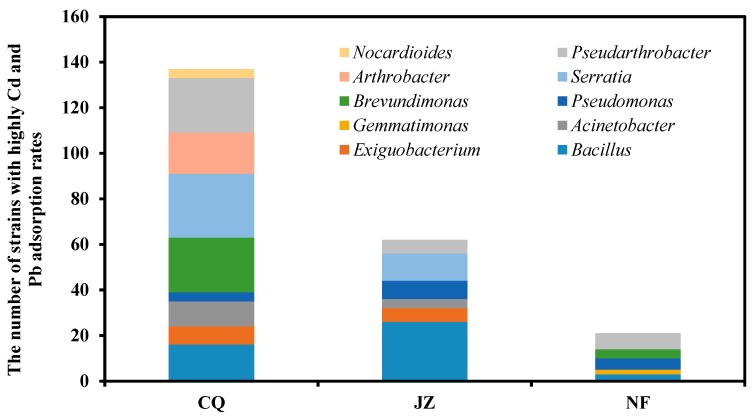
Genus-level differences of the heavy metal-immobilizing bacteria in CQ, JZ, and NF samples. CQ: farmland next to the company area; JZ: farmland near Jiazhuang village 3 km away from the company; NF: farmland near Nanfan village 6 km away from the company.

Heavy metal adsorption by culturable bacteria was examined in the CQ, JZ, and NF samples. The removal efficiency of heavy metals in the solution was determined by measuring the concentrations of Cd^2+^ and Pb^2+^ in the supernatant. We used a heavy metal removal efficiency greater than 80% as a cutoff, which indicated that the strain had strong heavy metal fixation and adsorption capacities. A removal rate of less than 80% indicated that the strain had a weak fixed adsorption capacity for Cd and Pb. The results are shown in Figure 3. Among the 400 culturable bacteria in the CQ samples, 137 had Cd^2+^ and Pb^2+^ adsorption rates (81.5–92.7%) over 80%, accounting for 34.3% of bacteria. Strains belonging to *Brevundimonas*, *Serratia*, and *Pseudoarthrobacter* had stronger abilities to fix Cd^2+^ and Pb^2+^, accounting for more than 20 strains. Among the 400 culturable bacteria in the JZ samples, 62 had Cd and Pb adsorption rates (82.7–91.1%) over 80%, accounting for 15.5% of bacteria. Strains belonging to *Bacillus* and *Serratia* had stronger abilities to fix Cd^2+^ and Pb^2+^. Among the 400 culturable bacteria in the NF samples, 21 had Cd^2+^ and Pb^2+^ adsorption rates (80.4–88.4%) over 80%, accounting for 5.2% of bacteria. This showed that soil bacteria evolve mechanisms to deal with the toxicity of heavy metals and carry out immobilization and passivation of heavy metals after colonizing the water spinach rhizosphere soil.

### 3.4. Growth Promotion Characteristics and Heavy Metal Resistance of Heavy Metal-Immobilizing Bacteria

The production of IAA and siderophores and MICs of Cd^2+^ and Pb^2+^ were determined for 221 bacteria with strong Cd^2+^ and Pb^2+^ immobilizing activities in the CQ, JZ, and NF samples. The results are shown in Table 2. Finally, 25 strains producing high levels of IAA (20.65–87.61 mg·L^−1^) and siderophores were screened. The highest IAA concentrations were found in *Enterobacter bugandensis* CQ-7, *Bacillus megaterium* CQ-30, *Bacillus thuringensis* CQ-33, *Klebsiella michiganensis* CQ-169, and *Serratia nematodiphila* JZ-262, which were 87.61, 69.65, 71.63, 58.63, and 55.27 mg L^−1^, respectively. All 25 strains showed strong resistance to Cd^2+^ (100–600 mg L^−1^) and Pb^2+^ (1200–2000 mg L^−1^). The MICs of Cd and Pb were 700 and 2200 mg L^−1^ for *Enterobacter bugandensis* CQ-7, 600 and 2200 mg L^−1^ for *Bacillus thuringensis* CQ-33, and 600 and 1800 mg L^−1^ for *Klebsiella michiganensis* CQ-169, respectively.

### 3.5. Effects of Heavy Metal-Immobilizing Bacteria on the Growth and Enrichment of Cd^2+^ and Pb^2+^ in Water Spinach

**Figure 4:**
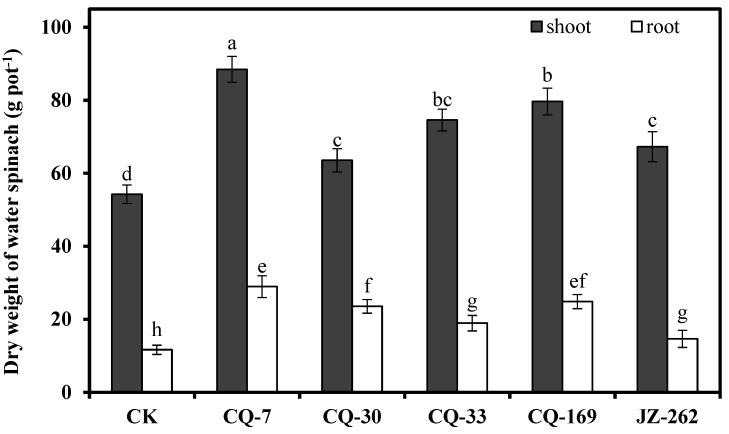
Effects of strains on the dry weight of water spinach in the 5 mg L^−1^ Cd^2+^ and 20 mg L^−1^ Pb^2+^ contaminated solution (x ± s). Error bars are ± standard error (*n* = 3). Bars indicated by the different letter (a-h) were significantly (*p* < 0.05) different according to Tukey’s test.

**Figure 5:**
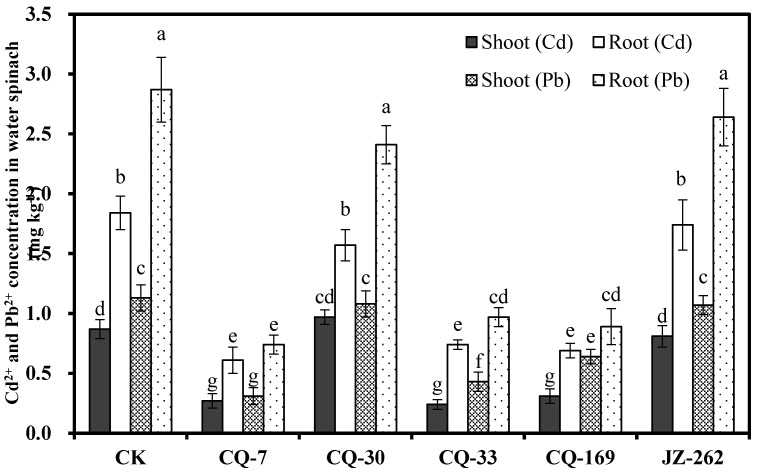
Effects of strains on Cd^2+^ and Pb^2+^ accumulation in water spinach in the 5 mg L^−1^ Cd^2+^ and 20 mg L^−1^ Pb^2+^ contaminated solution (x ± s). Error bars are ± standard error (*n* = 3). Bars with different letter (a–g) for were significantly (*p* < 0.05) different according to Tukey’s test.

Five heavy metal-immobilizing bacteria, *Enterobacter bugandensis* CQ-7, *Bacillus megaterium* CQ-30, *Bacillus thuringensis* CQ-33, *Klebsiella michiganensis* CQ-169, and *Serratia nematodiphila* JZ-262, which showed high production of IAA and siderophores and high resistance to Cd and Pb were used to study their effects on the growth and enrichment of Cd and Pb in water spinach. The results are shown in Figure 4 and Figure 5. Compared to the control, inoculation with these five strains significantly (*p* < 0.05) improved the dry weight of water spinach roots (25.75–148%) and shoots (17.16–63.14%), demonstrating that these five strains promoted the growth of water spinach. Among them, strain CQ-7 increased the dry weight of the root by 148% and the shoot by 63.14%; strain CQ-33 increased the dry weight of root by 62.66% and the shoot by 37.53%; and strain CQ-169 increased the dry weight of root by 133% and the shoot by 46.86%.

The contents of Cd^2+^ and Pb^2+^ in the shoots of water spinach were 0.87 and 1.13 mg kg^−1^ in the control group, respectively. Compared to the control, the contents of Cd^2+^ (59.78–72.41%) and Pb^2+^ (43.36–74.21%) in the roots and shoots of water spinach were significantly (*p* < 0.05) reduced by strains CQ-7, CQ-33, and CQ-169, whereas the contents of Cd^2+^ and Pb^2+^ in the roots and shoots of water spinach were not significantly changed by strains CQ-30 and JZ-262.

### 3.6. Effects of Strains on the Soluble Protein and Vc Contents of Water Spinach

**Figure 6:**
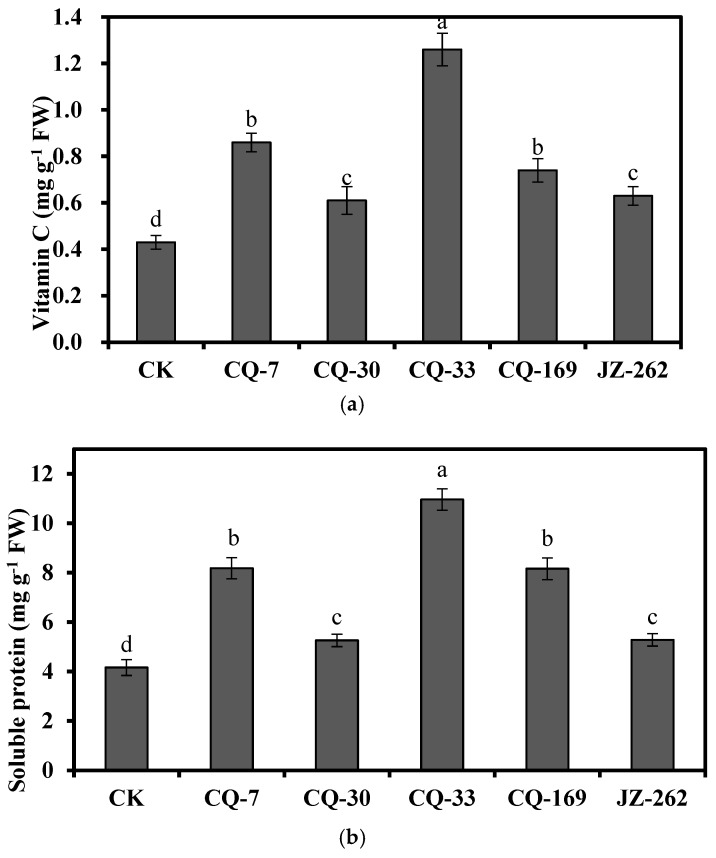
Effects of strains on the contents of solution protein and vitamin C in shoots of the water spinach grown in the 5 mg L^−1^ Cd^2+^ and 20 mg L^−1^ Pb^2+^ contaminated solution (x ± s). (**a**): Vitamin C content; (**b**): Soluble protein content. Error bars are ± standard error (*n* = 3). Values followed by different lowercase letters (a–d) significantly at the *p* < 0.05 level (Tukey’s test). FW: fresh weight.

The soluble protein content in the shoot of the water spinach was 4.16 mg g^−1^ fresh weight in the absence of the strains; however, the soluble protein content in the shoots was 5.26–10.96 mg g^−1^ fresh weight when inoculated with the strains CQ-7, CQ-30, CQ-33, CQ-169, and JZ-262 (Figure 6a). Compared with the control, inoculation with the strains CQ-7, CQ-30, CQ-33, CQ-169, and JZ-262 also increased the Vc content (46.5–193%) of the shoots (Figure 6b), indicating that the two strains improved lettuce quality. In addition, the soluble protein and Vc contents in the shoots were increased by 22.7–28.9% with strain HD8 compared to strain TJ6.

## 4. Discussion

### 4.1. High Concentration Heavy Metals Increased the Proportion of Heavy Metal-Immobilizing Bacteria in Water Spinach Rhizosphere Soil

The presence of microorganisms in soil is of great significance to the improvement of soil quality and biological activity [37]. Microorganisms are the main force in bioremediation of polluted soil and some functional microorganisms reduce the level of soil pollution [38]. Microbial communities are, additionally, also highly sensitive to environmental changes; therefore, they are considered indicators of local environmental conditions [26,39]. Heavy metals affect soil microorganisms mainly by altering the microbial community structure, reducing biomass, and affecting their biological activities [40]. The number of microorganisms in soil is very sensitive to increased heavy metal concentrations and plays an important role in nutrient cycling and ecosystem sustainability [41]. Yao et al. [42] showed that in the soil of a polluted mining area, the microbial biomass of soil near the Cu and Zn mine area was significantly lower than that distant from the mining area; sites closer to the mining area showed a more obvious decline in biomass. In this study, the number of culturable bacteria in the CQ sample (high concentrations of Cd^2+^ and Pb^2+^) was 6.1 × 10^3^ CFU g^−1^ soil, whereas those in the JZ and NF samples (low concentrations of Cd^2+^ and Pb^2+^) were 1.65 × 10^5^ and 1.28 × 10^6^ CFU g^−1^ soil, respectively, indicating that high concentrations of heavy metals inhibited the growth and reproduction of some bacteria and reduced the biomass of bacteria in the soil.

Heavy metal pollution not only reduces the biomass of soil microorganisms, but also affects the structure of the soil microbial community, including soil microbial diversity, species diversity, functional diversity, genetic diversity, and ecological characteristics diversity [43]. Although many scholars have studied that heavy metal stress influences the microbial community structure and function in soil [44], the relationship between the long-term polluted soil and the community structure of culturable microorganisms and heavy metal-immobilizing bacteria in the water spinach rhizosphere is still unclear. Our results demonstrated that the community structure of culturable bacteria in water spinach rhizosphere soil was altered in soil polluted with Cd and Pb. This indicates that the high concentration of heavy metals reduced the taxonomic units of culturable bacteria at the phylum and genus levels and reduced the diversity of culturable bacteria in the rhizosphere of water spinach. In general, the toxicity of heavy metals affects less-resistant microorganisms and reduces their abundances, such as Firmicutes and Bacteroidetes. Their abundances are negatively related to the concentration of Cd [45], whereas heavy metals increase the abundance of some metal-resistant bacteria [46]. In this study, among the 400 culturable bacteria in the CQ samples, 137 strains (34.3%) showed Cd^2+^ and Pb^2+^ adsorption rates over 80%, however, among the 400 culturable bacteria in the NF samples, 21 strains (5.2%) showed Cd^2+^ and Pb^2+^ adsorption rates over 80%, this indicates that the high concentration of heavy metals significantly increased the proportion of heavy metal-immobilizing bacteria in water spinach rhizosphere soil. Under conditions of high Cd and Pb pollution, *Acinetobacter*, *Serratia*, and *Bacillus* were identified as the dominant genera in heavy metal-polluted soil and play some specific roles in the resistance and fixation of heavy metals [47]. *β*-Proteobacteria is the most important phylum in water spinach rhizosphere soil with high Cd and Pb pollution levels. They have been recognized as the most important microorganisms in heavy metal-polluted soil in previous studies [23]. Wei et al. [48] found that Mn significantly affected the endophytic bacterial diversity and community structure of the leaves of *Phytolacca americana*, and its shannon index decreased significantly with increasing soil Mn concentration. Heavy metal-resistant bacteria increased with increasing heavy metal concentrations, whereas sensitive bacteria were decreased [49]. The reason for the change in the community structure of culturable bacteria in water spinach rhizosphere soil of the CQ, JZ, and NF samples in this study may be that some rhizobacteria with low resistance to heavy metals decreased or disappeared because they could not adapt to the high concentrations of Cd^2+^ and Pb^2+^, such as *Mucilaginibacter*, *Chryseobacterium*, *Terrabacillus*, and *Staphylococus*. Rhizobacteria with heavy metal resistance and fixation ability had competitive advantages and proliferated in large quantities; these bacteria included *Acinetobacter*, *Arthrobacter*, and *Pseudoarthrobacter*. In addition, to resist the toxic effects of Cd and Pb, crops select beneficial rhizosphere and endophytic bacteria.

### 4.2. Heavy Metal-Immobilizing Bacteria Inhibit the Absorption of Heavy Metals in Water Spinach

Crops grown in these polluted soils show heavy metal contents that exceed the standard limits, causing serious threats to human health [50]. Cd and Pb concentrations in the shoots and roots of water spinach were significantly lower in the treatments of CQ-7, CQ-33, and CQ-169 relative to the control group, suggesting that strains CQ-7, CQ-33, and CQ-169 could be used as a heavy metal passivator to reduce the absorption of Cd^2+^ and Pb^2+^ by water spinach. The passivation effect of heavy metal-immobilizing bacteria on heavy metals can cause the heavy metals to precipitate or be adsorbed and fixed in the soil, change the existing state of heavy metals, and “passivate” their bioavailability, thus reducing the absorption of heavy metals by crops [13]. Li et al. [21] reported that the heavy metal-immobilizing bacterium *Bacillus megaterium* H3 decreased Cd availability in the soil and Cd^2+^ content in rice. Banik et al. [51] reported that two plant-associated strains, *Bacillus aquimaris* SB1 and *Halobacillus truepri* SB2, reduced heavy metal accumulation and withstood metal and salinity stress in groundnut seedlings. Heavy metal-immobilizing bacteria immobilize heavy metals by adsorption to the cell wall, complexation with phosphate, or precipitation or adsorption reactions with heavy metal by other anions generated by bacterial metabolism [52]. Therefore, the ability to passivate heavy metals can be determined by measuring the adsorption capacity of the strains. In this study, 137 strains were isolated from CQ samples. This indicates that the strains reduced the total amount of Cd^2+^ and Pb^2+^ in the solution by adsorbing and thus reducing the contents of Cd^2+^ and Pb^2+^ in water spinach.

Resistance to heavy metals in bacteria is a prerequisite for using such bacteria to immobilize heavy metals. The mechanisms involved in bacterial heavy metal resistance are extracellular sequestration, intracellular sequestration, metal exclusion by a permeability barrier, enzymatic detoxification, and active transport of the metal [53]. The MICs of Cd^2+^ and Pb^2+^ for strain CQ-7 were 700 mg L^−1^ and 2200 mg L^−1^, and those for strain CQ-33, were 600 mg L^−1^ and 2200 mg L^−1^, and those for strain CQ-169, were 600 mg L^−1^ and 1800 mg L^−1^, indicating strains CQ-7, CQ-33, and CQ-169 could be used for efficient immobilization of heavy metals. The main function of plant growth-promoting bacteria is to promote plant growth, mainly by secreting arginine decarboxylase, siderophores, IAA, and other substances [54]. Singh et al. [55] reported that the plant growth-promoting bacterium NBRI012 produces IAA and increases the biomass of rice in As-contaminated soils. Saleem et al. [56] found that compared to control treatment, treatment with the plant growth-promoting bacteria *Pseudomonas fluorescens* S5 and *Pseudomonas gessardii* S2 improved sunflower growth and reduced the malondialdehyde content. In our study, strains CQ-7, CQ-33, and CQ-169 produced IAA and increased the root and shoot dry weights of water spinach compared to control values in the Cd- and Pb-contaminated soils. Our results demonstrated that strains CQ-7, CQ-33, and CQ-169 significantly promoted the edible tissue growth of the water spinach. In addition, strains CQ-7, CQ-33, and CQ-169 also significantly improved edible tissue quality (indicated by soluble protein and Vc contents) of the water spinach. Plant growth-promoting and heavy metal-resistant *Neorhizobium huautlense* T1-17 was also reported to increase edible tissue soluble protein and Vc contents of vegetables [30]. strains CQ-7, CQ-33, and CQ-169 demonstrated multiple plant growth-promoting characteristics which might be related to the protection of the water spinach against Cd and Pb toxicity and the edible tissue growth promotion in the presence of strains CQ-7, CQ-33, and CQ-169.

In this study, we focused on the effects of heavy metal-immobilizing bacteria on edible tissue growth and Cd^2+^ and Pb^2+^ uptake of the water spinach in metal-contaminated solution. Our results showed that edible tissue Cd^2+^ contents (ranging from 0.06 mg kg^−1^ to 0.11 mg kg^−1^ of fresh weight) and Pb^2+^ contents (ranging from 0.11 mg kg^−1^ to 0.19 mg kg^−1^ of fresh weight) in the shoots of water spinach met the standard for limit of Cd^2+^ (0.2 mg kg^−1^ of fresh weight) and Pb^2+^ (0.3 mg kg^−1^ of fresh weight) in vegetables in FAO (Food and Agriculture Organization of the United Nations)/WHO (World Health Organization) [57] in the presence of strains CQ-7, CQ-33 and CQ-169. These results provide evidence for the usefulness of strains CQ-7, CQ-33, and CQ-169 for heavy metal phytostabilization and subsequently decrease the risk of Cd^2+^ and Pb^2+^ entering the food chain.

## 5. Conclusions

In our study, three samples (CQ, JZ, and NF) of water spinach rhizosphere soils were collected. Compared to low-concentration Cd- and Pb-pollution (JZ and NF), high-concentration Cd^2+^ and Pb^2+^ pollution (CQ) significantly reduced the biomass of culturable bacteria and increased the proportion of heavy metal-immobilizing bacteria. The 400 bacteria in the CQ samples belonged to 3 phyla and 14 genera. *β-*Proteobacteria was the dominant phylum and *Acinetobacter*, *Brevundimonas*, *Serratia*, *Arthrobacter*, and *Pseudarthrobacter* were the dominant genera. Three heavy metal-immobilizing bacteria, *Enterobacter bugandensis* CQ-7, *Bacillus thuringensis* CQ-33, and *Klebsiella michiganensis* CQ-169 produced siderophores and IAA and were highly resistant to Cd^2+^ and Pb^2+^. Compared to the control, strains CQ-7, CQ-33, and CQ-169 significantly increased the dry weight of water spinach and reduced the contents of Cd^2+^ and Pb^2+^ in water spinach. The results showed the importance of heavy metal-immobilizing bacteria in vegetable growth and metal accumulation. The results also highlighted that the effectiveness of heavy metal-immobilizing bacteria-vegetable systems must be tested and established in controlled vegetation experimental designs with consideration of the specific matching of vegetables and bacteria. A further understanding of the mechanisms involved in the edible tissue growth, Cd and Pb availability and Cd^2+^ and Pb^2+^ accumulation of vegetables in the presence of strains CQ-7, CQ-33, and CQ-169 is needed for a practical strategy for the remediation and safe production of vegetables in metal-contaminated soils.

## Figures and Tables

**Table 1:** Physical and chemical properties of three water spinach rhizosphere soil samples (x ± s).

Sample	DTPA-Extractable Contents (mg kg^−1^)		pH	Organic Matter g·kg^−1^	Total P g·kg^−1^	Total N g·kg^−1^	Total K g·kg^−1^	Total Number of Bacteria CFU g^−1^
	Cd	Pb						
CQ	4.35 ± 5.3a	22.5 ± 2.67a	6.22 ± 0.03c	18.4 ± 1.1b	3.4 ± 0.28b	5.2 ± 0.3b	5.4 ± 0.52b	6.1 × 10^3^ ± 543c
JZ	1.62 ± 0.32b	11.7 ± 1.16b	6.42 ± 0.05b	25.4 ± 2.1a	6.1 ± 0.81a	7.1 ± 0.5b	7.5 ± 0.73a	1.65 × 10^5^ ± 1740b
NF	0.12 ± 0.03c	3.75 ± 0.21c	6.97 ± 0.04a	22.6 ± 1.7a	5.6 ± 0.32a	8.9 ± 0.2a	8.1 ± 0.34a	1.28 × 10^6^ ± 7854a

CQ: farmland next to the company area; JZ: farmland near Jiazhuang village 3 km away from the company; NF: farmland near Nanfan village 6 km away from the company. Error bars are ± standard error (*n* = 3). The presented data is represented by mean values and standard error. Data followed by the different letters (a–c) within the same column are significantly different (*p* < 0.05) according to Tukey’s test.

**Table 2:** Growth promotion characteristics and heavy metal resistance of heavy metal-immobilizing bacteria.

Strain	MICs of Cd^2+^ (mg L^−1^)	MICs of Pb^2+^ (mg L^−1^)	IAA (mg L^−1^)	Siderophores
CQ-2	200	1500	23.65 ± 1.87	+++
CQ-7	700	2200	87.61 ± 4.32	+++++
CQ-8	300	1800	33.57 ± 2.12	++
CQ-12	300	1500	49.65 ± 2.89	+++
CQ-19	400	1500	38.64 ± 2.23	++
CQ-30	400	1800	69.65 ± 3.57	+++++
CQ-33	600	2200	71.63 ± 3.99	+++++
CQ-39	300	1800	33.54 ± 1.85	+++
CQ-53	200	1500	29.68 ± 1.64	++
CQ-59	300	1800	45.87 ± 2.46	++
CQ-83	400	1500	32.85 ± 1.87	+++
CQ-123	300	1500	42.98 ± 2.14	+++
CQ-169	600	2000	58.63 ± 2.76	+++++
CQ-203	400	1800	48.35 ± 2.43	+++
CQ-243	300	1500	29.64 ± 1.34	++
CQ-295	500	1500	54.28 ± 2.54	+++
CQ-363	200	1800	34.75 ± 1.56	+++
JZ-17	200	1500	54.18 ± 2.65	++
JZ-33	300	1500	28.64 ± 1.45	+++
JZ-68	200	1500	65.27 ± 3.56	++
JZ-83	200	1500	38.54 ± 2.45	+++
JZ-163	300	1800	48.61 ± 2.45	++
JZ-262	400	1800	55.27 ± 3.12	++++
JZ-263	200	1500	23.67 ± 1.32	+++
JZ-311	200	1500	44.75 ± 2.11	+++

MICs: minimal inhibitory concentrations; IAA: indole-3-acetic. Error bars are ± standard error (*n* = 3). More’+’ in the fifth column indicates more siderophores the strain can produce.
